# KL-6 in ANCA-Associated Vasculitis Patients with and without ILD: A Machine Learning Approach

**DOI:** 10.3390/biology11010094

**Published:** 2022-01-08

**Authors:** Edoardo Conticini, Miriana d’Alessandro, Laura Bergantini, Diego Castillo, Paolo Cameli, Bruno Frediani, Luca Cantarini, Elena Bargagli

**Affiliations:** 1 Rheumatology Unit, Department of Medicine, Surgery & Neurosciences, University of Siena, 53100 Siena, Italy; conticini.edoardo@gmail.com (E.C.); fredianibruno60@gmail.com (B.F.); cantarini@unisi.it (L.C.); 2 Respiratory Diseases and Lung Transplantation Unit, Department of Medical and Surgical Sciences & Neurosciences, University of Siena, 53100 Siena, Italy; laurabergantini@gmail.com (L.B.); paolocameli88@gmail.com (P.C.); bargagli2@gmail.com (E.B.); 3 Respiratory Department, Hospital de la Santa Creu i Sant Pau, Sant Pau Biomedical Research Institute (IIB-Sant Pau), 08041 Barcelona, Spain; dcastillo@santpau.cat

**Keywords:** KL-6, ANCA-associated vasculitis, interstitial lung disease

## Abstract

**Simple Summary:**

Krebs von den Lungen-6 (KL-6) is a mucin involved in several cellular processes, and its expression increases following injured or regenerating type II pneumocyte. KL-6 was suggested to identify patients affected by fibrotic interstitial lung diseases (ILD) associated with rheumatologic disorders. This is the first study that has assessed whether serum KL-6 can distinguish ANCA-associated vasculitis (AAV) patients with ILD from those without ILD, and whether this biomarker and its changes over time are correlated with disease activity, vasculitic damage, and response to treatment. Thirteen AAV patients were enrolled, four of them with ILD. Higher serum KL-6 was found in AAV-ILD patients in comparison with those without ILD. The best KL-6 cutoff values of 368 U/mL and 301 U/mL at baseline and during follow-up, respectively, were suggested to distinguish the two groups. A direct correlation was found between serum KL-6 and disease activity. Our multicenter study demonstrated KL-6 as a reliable, non-invasive, and easy-to-perform marker of ILD in AAV patients, being helpful for disease activity assessment. Changes in serum concentrations of KL-6 over time could be useful for monitoring AAV patients. Further study of KL-6 as a marker of response to therapy during long-term follow-up would also be worthwhile.

**Abstract:**

Background: ANCA-associated vasculitis (AAV) are small vessel vasculitis distinguished between microscopic polyangiitis (MPA) and granulomatosis with polyangiitis (GPA). The former may have interstitial lung disease (ILD) associated with high morbidity and mortality. Here, Krebs von den Lungen-6 (KL-6), a marker of fibrotic ILD, was assessed for distinguishing AAV patients with ILD from those without ILD, and whether its changes over time are correlated with disease activity. Materials and Methods: Thirteen AAV patients (eight females, mean age 61 ± 14.8 years) were enrolled: six MPA and six GPA. Serum samples were assayed for KL-6 concentrations (Fujirebio Europe, Belgium). To investigate potential binary classifiers for diagnosis of AAV-ILD, we constructed a regression decision tree model. Results: Higher serum KL-6 were in AAV-ILD compared with those without ILD (972.8 ± 398.5 vs 305.4 ± 93.9, *p* = 0.0040). Area under the receiver operating characteristics curve showed 100% of the diagnostic performance of KL-6 for identifying the ILD involvement (accuracy 91.7%) and the best cutoff value of 368 U/mL (sensitivity 100% and specificity 87.5%). The decision tree model showed a 33% improvement in class purity using a cut-off value of 513 U/mL to distinguish AAV patients with and without ILD. Stratifying AAV patients as MPA and GPA with and without ILD considering T0 and T1 KL-6, the model obtained an improvement of 40% for classifying GPA non-ILD with a T0 serum KL-6 cut-off value of 513 U/mL and a T1 KL-6 cut-off of 301 U/mL. A direct correlation was found between serum T0 KL-6 and T0 BVAS (r = 0.578, *p* = 0.044). Conclusion: Our multicenter study demonstrated KL-6 as a reliable, non-invasive, and easy-to-perform marker of ILD in AAV patients and its helpfulness for disease activity assessment. Changes in serum concentrations of KL-6 over time could be useful for monitoring AAV patients. Further study of KL-6 as a marker of response to therapy during long-term follow-up would also be worthwhile.

## 1. Introduction

ANCA-associated vasculitis (AAV) are small vessel vasculitis affecting the kidneys, upper and lower airways, skin, and central and peripheral nervous systems [1]. They are distinguished between microscopic polyangiitis (MPA), granulomatosis with polyangiitis (GPA), or eosinophilic granulomatosis with polyangiitis (EGPA), which have different signs, symptoms, and autoimmune profiles. MPA is usually associated with ANCA-MPO positivity and GPA with ANCA-PR3 positivity, whereas up to 50% of patients with EGPA are ANCA-negative [2,3].

Patients with MPA, especially those who are ANCA-MPO-positive, may have interstitial lung disease (ILD) [4,5], which is associated with high morbidity and mortality as it is often underdiagnosed and responds poorly to conventional treatments [6]. Interstitial lung disease associated with AAV does not have specific imaging or bronchoalveolar lavage (BAL) findings. Moreover, due to lack of validated diagnostic criteria [7], the diagnosis of AAV relies on clinical expertise and often requires invasive biopsy procedures.

In the lung, Krebs von den Lungen-6 (KL-6) is a key transmembrane mucin implicated in the process of cell proliferation, growth, and apoptosis; its expression increases in injured or regenerating type II pneumocytes [8,9,10,11]. KL-6 was recently proposed as a diagnostic biomarker for ILD and for predicting response to antifibrotic therapies [12,13]. Different reports have demonstrated elevated serum KL-6 concentrations in various ILDs, including connective tissue disorders associated with ILD [14,15,16,17], suggesting that this marker is not only useful for diagnosis but also for prognosis and for monitoring response to therapy.

Some authors have reported higher serum concentrations of KL-6 in AAV patients with ILD than in those without lung involvement, suggesting that this mucin could be a useful prognostic marker [18,19,20]. However, these few studies were conducted in small cohorts and did not consider disease activity or extrapulmonary manifestations.

Our aim here was therefore to assess whether serum KL-6 can distinguish AAV patients with ILD from those without ILD, and whether this biomarker and its changes over time are correlated with disease activity, vasculitic damage, and response to treatment.

## 2. Materials and Methods

### 2.1. Study Population

We enrolled all consecutive patients assessed at the Vasculitis Clinic of the Rheumatology and Respiratory Disease Units of Siena University Hospital in the period December 2020 to November 2021 and ILD clinic of Hospital de la Santa Creu I Sant Pau (Barcelona) in the period 2017 to 2018. Inclusion criteria were granulomatosis with polyangiitis (GPA) or microscopic polyangiitis (MPA) diagnosed by a clinician with experience in vasculitis, active disease, and eligibility for rituximab treatment according to current recommendations [21].

Exclusion criteria were a diagnosis of EGPA and remitting disease. As part of our clinical practice, all patients underwent concomitant rheumatological and pneumological evaluation, lung function tests, routine blood tests, autoimmunity evaluation, and KL-6 assay. Current and previous treatments, Birmingham vasculitis score (BVAS), and vasculitis damage index (VDI) were also recorded. High-resolution computed tomography (HRCT) and BAL were performed if lung involvement was suspected.

The first evaluation in the observation period at our vasculitis clinic (Siena) and ILD clinic (Barcelona) was defined as baseline (T0); the medical examination after treatment was defined as T1.

Serum samples from all patients were assayed for KL-6 concentrations by KL-6 reagent assay (Fujirebio Europe, United Kingdom), as previously reported [14]. The principle of the assay is agglutination of sialylated carbohydrate antigen in samples with KL-6 mAb by antigen–antibody reaction. The change in absorbance was measured to determine KL-6 levels. Serum concentrations of KL-6 were expressed in U/mL. A serum KL-6 cut-off value of 465 U/mL was considered to distinguish fibrotic-ILD patients from non-fibrotic patients and healthy subjects, as previously reported [22].

All patients gave their written informed consent to participate in the study, which was approved by our local ethics committee (Siena, Markerlung 17431 and Barcelona, IIBSP-KLS-2016-39).

### 2.2. Statistical Analysis

The data are presented as mean ± standard deviation. Non-parametric one-way ANOVA (Kruskal–Wallis test) and Dunn’s test were performed for multiple comparisons. Wilcoxon’s rank sum test was used to compare data before and after treatment (T0–T1). Receiver operating characteristics (ROC) curve was employed to analyze the diagnostic performance of KL-6 for identifying the ILD involvement and to select the best cutoff threshold with high sensitivity and specificity.

In order to investigate potential binary classifiers for diagnosis of AAV-ILD, we formed a group of patients with AAV but not ILD. Then, we constructed a regression decision tree for AAV with versus without lung involvement, MPA-ILD versus GPA non-ILD and MPA non-ILD, and previous versus non-previous treatment. We created a series of test/training partitions to evaluate the accuracy of potential binary classifiers by means of a confusion matrix. The best thresholds, in terms of specificity and sensitivity, of each binary classifier were chosen by Youden’s J method. The Spearman test was used to look for correlations. A *p*-value less than 0.05 was considered statistically significant. Statistical analysis was performed by GraphPad Prism 9.2 and XLSTAT 2021 software.

## 3. Results

Demographic, immunological, and clinical data are shown in Table 1, together with BVAS, VDI, and organ involvement. A total of 13 patients (8 females, 5 males, median age 61 ± 14.8 years) were enrolled. One was excluded due to a concomitant diagnosis of lung cancer. Six had MPA and the other six had GPA; median duration of disease was 4.8 ± 6.5 years. Serum concentrations of KL-6 in AAV patients with ILD were compared with those of AAV patients without ILD (972.8 ± 398.5 vs 305.4 ± 93.9, *p* = 0.0040).

**Figure 1:**
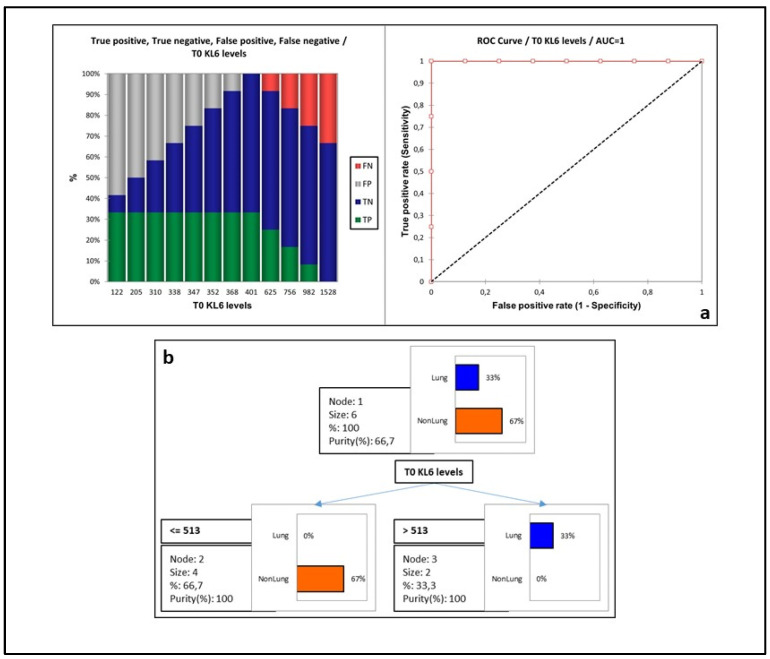
(**a**) Receiver operating characteristic (ROC) curves were first employed to analyze the diagnostic performance of KL-6 for identifying the ILD involvement and to select the best cutoff threshold with high sensitivity and specificity. (**b**) Decision tree model obtained using KL-6 concentrations at T0 showed a 33% improvement in class purity using a cut-off value of 513 U/mL to distinguish AAV patients with and without ILD (*p* = 0.034). Lung and non-lung are referred to the presence and absence of ILD, respectively. Abbreviations: TP, true positive; TN, true negative; FP, false positive; FN, false negative.

ROC curve analysis between ILD and non-ILD in AAV patients (Figure 1a) showed the area under curve (AUC) of 100% with the best cut-off value of 368 U/mL (sensitivity 100% and specificity 87.5%, accuracy of 91.7%, LR+ 8.0 and LR- 0.0). In order to improve predictive power, we used a decision-tree model (with cross-validation by confusion matrix). The model (Figure 1b) obtained using KL-6 concentrations at T0 showed a 33% improvement in class purity using a cut-off value of 513 U/mL to distinguish AAV patients with (lung) and without (non-lung) ILD (*p* = 0.034).

**Figure 2:**
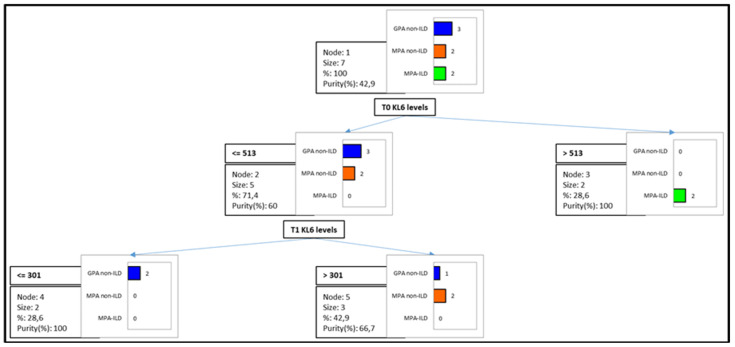
A decision tree model (and cross-validation by confusion matrix) was applied to T0 and T1 KL-6 concentrations in AAV patients stratified as MPA and GPA with and without ILD. The model obtained an improvement of 57.1% for classifying MPA-ILD patients with a serum T0 KL-6 cut-off of 513 U/mL and 40% of improvement for classifying GPA non-ILD with a T0 serum KL-6 cut-off value of 513 U/mL and a T1 KL-6 cut-off of 301 U/mL.

As expected, a higher prevalence of ILD was found in the MPA (20%) than in the GPA (10%) group. A decision tree model (and cross-validation by confusion matrix) was applied to T0 and T1 KL-6 concentrations in AAV patients stratified as MPA and GPA with and without ILD. The model (Figure 2) obtained an improvement of 57.1% for classifying MPA-ILD patients with a serum T0 KL-6 cut-off of 513 U/mL and 40% of improvement for classifying GPA non-ILD with a T0 serum KL-6 cut-off value of 513 U/mL and a T1 KL-6 cut-off of 301 U/mL.

**Figure 3:**
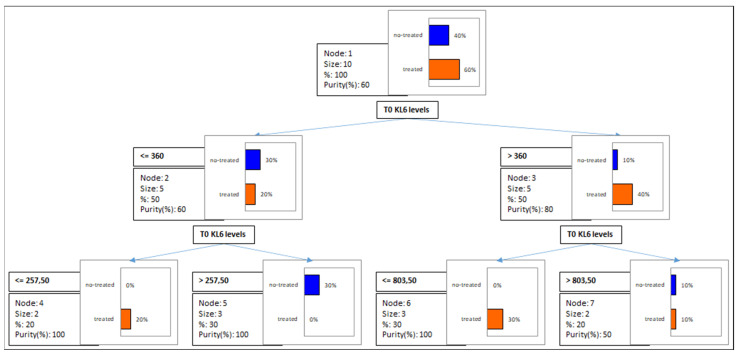
Decision tree model obtained from KL6 concentrations in AAV patients stratified according to previous treatment: 20% improvement in class purity for treated patients with a cut-off value of 360 U/mL and also with a further cut-off of 803.5 U/mL. The non-treated group used for the model obtained a 40% improvement in purity with a serum KL6 cut-off value of 257.5 U/mL.

Previous treatment did not influence serum concentrations of KL-6, as demonstrated by the decision tree model (and cross-validation by confusion matrix) (Figure 3), obtaining a 20% improvement in class purity for treated patients with a cut-off value of 360 U/mL and also with a further cut-off of 803.5 U/mL. The non-treated group used for the model obtained a 40% improvement in purity with a serum KL6 cut-off value of 257.5 U/mL.

**Figure 4:**
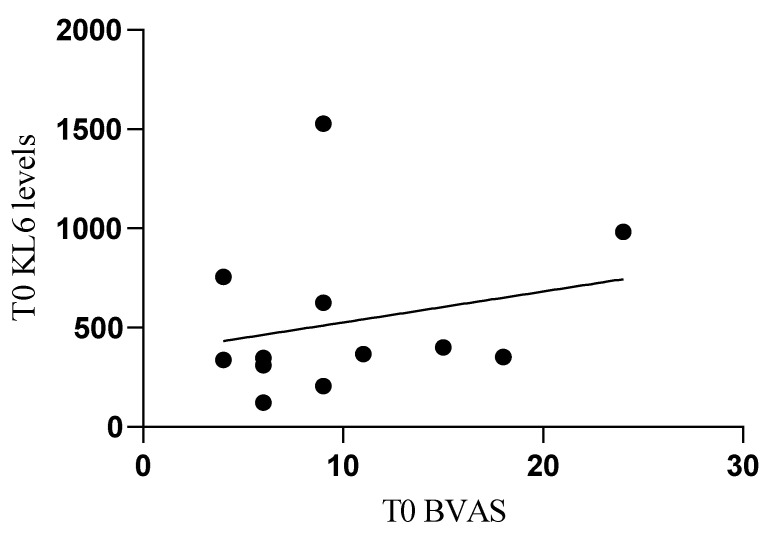
The correlation between serum concentrations of KL-6 at T0 and T0 BVAS (r = 0.578, *p* = 0.044) in AAV patients.

For clinical parameters, a direct correlation was found between serum concentrations of KL-6 at T0 and T0 BVAS (r = 0.578, *p* = 0.044) (Figure 4).

## 4. Discussion

This is the first time that serial measurements of serum KL-6 have been made in AAV patients in order to assess the utility of this glycoprotein for diagnosis, prognosis, and monitoring of these patients. Respiratory tract involvement in AAV may be extremely varied [23], ranging from diffuse alveolar hemorrhage (DAH), typically occurring in MPA, to nodules, usually found in GPA, and eosinophilic pneumonia, which together with asthma and transient infiltrates is the hallmark of EGPA.

Although not considered a typical feature of AAV, ILD is reported in a certain percentage of patients: up to 45% and 23% of MPA and GPA patients, respectively, have various forms of ILD [4], while only one case of EGPA and pulmonary fibrosis is reported in the literature. In line with these previous observations, interstitial lung involvement in our cohort was generally detected in MPA patients: significantly, the only GPA patient with concomitant ILD was ANCA-MPO-positive.

ILD usually occurs months to years before diagnosis of AAV. Occasionally, the diagnosis of AAV is concomitant or precedes that of ILD.

ANCA themselves seem related to the development of ILD, even without concomitant vasculitis: several papers have reported a significant variable percentage of ANCA-positive patients with ILD but with no extra-pulmonary signs or symptoms of AAV [24].

To date, there is no clear-cut diagnostic workup for this condition, and no imaging technique or laboratory analysis seems capable of predicting the onset of AAV-ILD or how it will respond to treatment. Among diagnostic procedures, HRCT has a well-established role, but as it exposes the patient to a significant dose of ionizing radiation and it is less suitable for close follow-up of patients with no definite ILD at baseline.

KL-6 is a high molecular weight glycoprotein, originally suggested as a serum biomarker for lung, breast, and pancreatic cancers, albeit with low diagnostic accuracy. Serum concentrations of KL-6 were found to be elevated in ILDs characterized by alveolar epithelial cell damage and progressive interstitial thickening [10]. A cut-off value of 465 U/mL was recently established to distinguish fibrotic ILD patients from healthy subjects and patients with other non-fibrotic lung diseases [22].

Elevated serum concentrations of KL-6 have been found in idiopathic pulmonary fibrosis (IPF) patients, a disease characterized by alveolar epithelial cell damage and progressive interstitial thickening. A prognostic role of KL-6 was suggested to predict survival in IPF patients evaluating its serum concentration at disease onset (cut-off 1000 U/mL) [25]. Fluctuations in KL-6 during follow-up of IPF patients have also been reported to have potential for predicting functional progression of the disease [26,27,28,29].

Only two studies have analyzed KL-6 concentrations in AAV patients with and without ILD, and one was a case report: both used an ELISA kit and neither considered overall disease activity or extra-pulmonary manifestations.

In line with these data, our multicenter study, which included eight female and five male patients (in line with most recent epidemiologic data [30]), confirmed the higher serum concentrations of KL-6 in AAV patients with ILD compared to those without ILD. On the other hand, no significant correlation was found for KL6 and extrapulmonary manifestations of vasculitis, or for lung involvement other than ILD. Likewise, KL6 concentrations did not appear to be higher in patients with a longer duration of disease or in those with multi-systemic involvement: this also confirms the high specificity of the biomarker in patients with complex, long-standing vasculitis who are more difficult to assess. At the same time, we found that previous pharmacological treatment did not influence elevated concentrations of KL-6 in AAV-ILD patients.

Similarly, no correlation was found between serum KL6 and VDI, which is not surprising, since lung fibrosis, chronic breathlessness, and impaired lung function are only 3 out of 63 items of this index. Conversely, and more importantly, a direct correlation was demonstrated between serum concentrations of KL6 at T0 and BVAS at T0. This confirms the strong link between KL6 and disease activity, which is of paramount importance as biomarker needs to be an easy diagnostic and prognostic tool, not merely useful for assessing organ or system damage.

The present study suggests a KL-6 cut-off value below 513 U/mL of serum, which may be particularly useful in patients in whom clinical (such as GPA) and serological features are not strongly associated with the onset of ILD.

Our study has some limitations: First, there was a limited number of patients who underwent KL-6 measurements over time that presumably underpowered its specificity at follow-up. Moreover, we were not able to establish a definite diagnostic cut-off value due to the limited sample size of patients with the concomitant ILD. Nevertheless, considering the rarity of disease and the relevant diagnostic issues due to the lack of definite imaging or laboratory findings for this condition, the demonstration of elevated serum concentrations of KL-6 in AAV patients with ILD is of interest. Further research lines should be addressed in order to assess whether KL-6 changes may be related to disease clinical course or response to treatment.

## 5. Conclusions

In conclusion, KL-6 was demonstrated to be a reliable, non-invasive, and easy-to-perform marker of interstitial lung involvement in AAV patients, being helpful for assessment of disease activity. Changes in serum concentrations of KL-6 over time could be useful for monitoring AAV patients. Further study of KL-6 as a marker of response to therapy during long-term follow-up would also be worthwhile.

## Figures and Tables

**Table 1:** Clinical, immunological, and demographic data of AAV patients. Abbreviations: AZA: azathioprine; BVAS: Birmingham vasculitis activity scale; F: female; GCs: glucocorticoids; GPA: granulomatosis with polyangiitis; KL6: Krebs von den Lungen; M: male; MMF: mycophenolate mofetil; MPA: microscopic polyangiitis; MTX: methotrexate; PNS: peripheral nervous system; RTX: rituximab; VDI: vasculitis damage index; * at T0.

Sex/Age	Diagnosis	Length of Disease * (months)	Organs Involved	Type of Lung Involvement	Previous Treatments	T0 Treatment	T0 GCs Dosage	T0 KL6 Levels	T0 BVAS	T0 VDI	T1 Treatment	T1 GCs Dosage	T1 KL6 Levels	T1 BVAS	T1 VDI
F/83	MPA	28	Lung, kidney, PNS	ILD, alveolar hemorrhage	RTX, AZA	GCs	10	625	8	7	RTX	5	325	0	7
M/77	MPA	8	Lung, kidney, PNS, skin	ILD, alveolar hemorrhage	-	GCs, MTX	15	982	24	1	-	-	-	-	-
M/71	GPA	24	Lung, eye, joints	Nodules, ILD	RTX	GCs	5	1528	9	3	-	-	-	-	-
F/64	MPA	24	Lung	ILD	-	-	-	756	4	*-*	-	-	1003	0	-
M/48	GPA	1	Lung, kidney, nose, eye	Nodules	-	GCs	50	352	18	0	RTX	25	279	2	0
F/55	GPA	252	Lung, skin	Nodules	CYC, MMF, AZA	GCs, MTX	25	401	15	3	GCs, RTX	12.5	602	3	3
F/38	MPA	1	Skin	-	-	-	-	347	6	0	-	-	-	-	-
F/74	MPA	180	Kidney, PNS	-	CYC	GCs	2.5	368	11	5	GCs, MTX	5	414	0	5
F/49	GPA	60	Nose, eye	-	MTX	GCs, RTX, AZA	5	122	6	2	GCs, RTX, AZA	5	116	0	2
M/60	MPA	40	Skin, PNS	-	GCs	MTX	-	205	9	1	-	-	-	-	-
F/39	GPA	4	Nose	-	-	GCs	50	310	6	3	-	-	-	-	-
F/59	GPA	72	Lung	Nodules, CT consolidation, biopsy confirmed GPA	-	-	-	338	4	*-*	-	-	-	0	-

## Data Availability

The data presented in this study are available on request from the corresponding author.

## Abbreviations

AZA	azathioprine
BVAS	Birmingham vasculitis activity scale
F	female
GCs	glucocorticoids
GPA	granulomatosis with polyangiitis
KL6	Krebs von den Lungen
M	male
MMF	mycophenolate mofetil
MPA	microscopic polyangiitis
MTX	methotrexate
PNS	peripheral nervous system
RTX	rituximab
VDI	vasculitis damage index
*	at T0
